# What future are you talking about? Efficacy of Life Design Psy-Lab, as career guidance intervention, to support university students’ needs during COVID-19 emergency

**DOI:** 10.3389/fpsyg.2022.1023738

**Published:** 2023-01-04

**Authors:** Elisabetta Camussi, Daria Meneghetti, Maria Luisa Sbarra, Riccardo Rella, Paolo Grigis, Chiara Annovazzi

**Affiliations:** ^1^Department of Psychology, University of Milan-Bicocca, Milan, Italy; ^2^Department of Human and Social Sciences, University of Valle D’Aosta, Aosta, Italy

**Keywords:** Life Design, soft skills, COVID-19, career guidance, University, Life satisfaction, Future

## Abstract

The pandemic increased world’s uncertainty, and led to deep change in daily habits, generating disorientation, and inevitably affecting people life and professional plans. Young adults and students’ future perspective and satisfaction with life were widely affected, especially for those facing school/ work transitions. Nevertheless, it was of primary importance for career guidance psychologists to intervene to support students despite having to navigate “on sight” and with little literature available. This paper will thus describe how the Life Design Psy-Lab, a career guidance service from Bicocca University (Milan, Italy) implemented a thematic online group intervention with 103 students enrolled. The intervention was based on the theoretical model of Life Design and was performed by career counselling psychologists. The intervention’s goal was to foster students’ development of skills and mindset to face complexity and unpredictability, shifting their time perception from an uncertain present to a more optimistic future scenario. The intervention’s impact was assessed using pre and post online questionnaires. Results show how students increased their precepted level in a set of skills to face change and complexity after participating to the intervention. In more detail, students’ precepted levels of career adaptability, courage, time perspective and resilience rose. Also, data show how the intervention strengthened students’ life satisfaction. This paper will thus contribute to expand the growing yet still newborn set of knowledge about career guidance interventions in times of COVID-19 emergency, specifically addressing the issue of design interventions that can improve young adults’ skills to visualize and project present and future for themselves, especially during uncertain times.

## 1. Introduction

The outbreak of the COVID-19 pandemic all over the world forced several changes in individuals’ lives. Despite being relatively safe about Covid’s medical issues, the youngest portion of world’s population appears to be deeply affected by the pandemic’s social and psychological outcomes. According to UNESCO (2020), more than 87% of the world’s student population was affected by the switch to remote teaching or mixed didactics. As UNESCO National Commission survey about the consequences of the pandemic on higher education found out, students dealing with transitions from school to university or from university to the job market face particular challenges. The significant damages caused by the pandemic resulted in a growth in global unemployment. Consequently, opportunities for university graduates to enter the labor market and start a professional career were narrowed (International Labour Organization, 2021). These topics were relevant to young adults even before the pandemics. However, COVID-19 can be considered a “career shock” for tertiary education students, widely affecting their sense of stability about the future and jeopardizing their ability to imagine and act proactively in building satisfying career trajectories (Akkermans et al., 2020). Recent findings about students during the lockdowns in Italy showed how they felt stuck in time, “aware of the competencies given by the years of training and university courses, but without knowing how and where to direct them” (Parola, 2020). Moreover, the lockdown measures fueled a sense of isolation between students who faced high levels of uncertainty, the impossibility to relate to peers, and disorientation. Daniel (2020) stated, “In many cases, they have been torn away from their social group almost overnight.” Other studies showed an increase in uncertainty intolerance indexes and perception of fear (Glowacz and Schmits, 2020), two significant risk factors affecting the ability to explore future scenarios with an optimistic view. In fact, these abrupt transformations and exposure to risks had a profound impact on students’ life paths, personal growth, and proactivity. It ultimately influenced their long-term ability to design and visualize possible career choices, opportunities, and options (Mahmud et al., 2021). The challenges university students face contributing to an increasing perception of loss of control, making them more focused on present concerns and susceptible to the avoidance of formative and occupational responsibilities. Indeed, uncertainty about the future obstructs the ability to develop successful strategies to manage career and educational planning, making students more vulnerable and less proactive toward self-construction (Holman and Grisham, 2020).

Since the outbreak of COVID-19 pandemic, studies in many sectors begun to flourish, to publish and disseminate both information on the situation in different contexts and good practices of intervention to manage and contrast the various consequences of this unprecedented global threat. With regards to Italy, Cattivelli and Rusciano (2020), for example, addressed the topic of social innovation applied to food accessibility in rural areas, demonstrating the importance of sharing common commitment and having clear purpose for improving wellbeing. Also, Rusciano and Gatto (2022) examined the role of the presence of Metropolitan Agricoltural Parks both in southern and northern Italy, identifying their role in population’s resilience and sustainability. With regards to interventions on young adults during the COVID-19 pandemics, there was, both nationally and internationally, great attention paid on mental health maintenance or improvement. Good view about this literature is provided by a systematic review by Riboldi et al. (2022), which examined different interventions prompted at enhancing university students’ mental health. These authors found that online cognitive behavioral therapy, dialectical behavior therapy, and mind–body practice techniques emerged as valid strategies, while digital positive psychology and mindfulness-based interventions showed mixed results. As regards for university students in the Italian context, Gabrielli et al. (2021) assessed the efficacy of an intervention for promoting healthy coping strategies, using a chatbot. Celia et al. (2022), instead, verified the effects of online counselling in enhancing positive emotions and diminishing anxiety and negative emotions. Finally, Mok et al. (2021) conducted a critical review about the skills needed by undergraduate students to prepare themselves for uncertain futures, in the view of a post-covid world in the middle of a fourth industrial revolution. They concluded that higher university institutions need to search for flexible approaches, in order to prepare students to manage uncertain futures, with a specific focus on soft skills.

After this paragraph the purpose of our study will be depicted, as long as its theoretical framework, namely the Life Design perspective and a set of skills proven to be extremely valuable in facing complex and uncertain times for young adults. Next, the career guidance service will be contextualized, as long as the intervention itself and the methodology used. Lastly, results, discussion, limits and future perspectives will be depicted.

## 2. Purpose of the study

The purpose of the present study is to assess the efficacy of an online Life Design career guidance intervention. The intervention was provided to a group of University of Milan-Bicocca’s students by the Life Design Psy-Lab, one of Bicocca’s career guidance services. Taking into consideration the uncertain and changing context described in the previous paragraph and the necessity to respond to the consequent vocational and professional needs of young people and adults, the intervention’s aim was the development of students’ personal skills to face and overcome uncertainty and complexity (career adaptability, resilience, courage and time perspective, as long as their life satisfaction). Examining this intervention’s outcome can on the one hand expand our knowledge about career guidance interventions in times emergency, in this case specifically related to COVID-19. On the other hand, to address the specific issue of improving young adults’ skills to visualize and project present and future for themselves, especially during uncertain times. A pre-post online questionnaire was used to assess the efficacy of the career guidance intervention in promoting student’s skills and satisfaction. The hypothesis is that students enrolled in the intervention will improve their precepted level in the skills considered, as long as their satisfaction with their life. Previously to the extensive description of the methodology used and the results obtained, the following paragraphs will explicate the choice of Life Design career guidance approach, the role of the skills promoted by the intervention, and the Life Design Psy-Lab career guidance service aims and focus.

## 3. Life design career guidance

The ongoing transformation of the relationship between individuals, institutions, and organizations requires an increasing ability to adapt and construct multiple career trajectories as a strategy to face uncertainty and design a professional path that sustains the individual’s wellbeing. The actual scenario, mixing the previous global complexity with the psychological consequences of the pandemic, is the ultimate challenge for professionals working in career guidance. The main goal is “to rekindle a sense of purpose and hope in people and devise strategies to convert hope into acting” (Maree, 2021). The actual situation highlighted the need for a re-adaptation of students’ ability to cope with the pandemic’s consequences and skills to meet the ongoing changes and characteristics of the new labor market, focusing on fluid working environments, practices, and conditions (UNESCO, 2020). Answering these needs, entangling different aspects of one’s life, needs a holistic and complex approach. Life Design is a career counselling theoretical and practical approach promptly aimed at fostering people’s adaptability in contexts of extreme uncertainty, non-linearity, and crisis (Savickas et al., 2009). Life Design was born at the beginning of the 2000s as a non-reductionist answer to new career guidance needs emerging from a more complex world. In the past modern era, finding a good, unique, and everlasting match between a person and a job was a satisfactory answer to personal and societal needs. Indeed, in a static and more predictable world, a person could retain a job placement for his or her whole life, worrying less about unexpected and disruptive changes. In the last decades, this paradigm has been showing its limits. Globalization, new technologies and communication, and the fragmented, ultra-specialized, ever-changing job world require flexible people. Readiness for an “ordinary unexpected” became the new “right person for the right place” (Taylor, 1914). This proved even more evident and fundamental with the advent of COVID-19. As stated, Life Design was raised as an answer to the guidance needs of people living in a world reshaping itself from its fundaments. With the radical and profound changes in everyone’s life brought by COVID-19, the Life Design paradigm found an ideal match, given its epistemological premises and methodological implications (Di Maggio et al., 2021; Maree, 2021). Therefore, Life Design career guidance supports people facing complexity, fostering their ability to adapt and be ready to anticipate and face change and unpredictability (Wen et al., 2020). That being said, career guidance interventions in the Life Design perspective start from reconstructing individuals’ personal stories and experiences. Life Design helps students shift their time perception, developing awareness about future trajectories and paths from individual narratives and values of self-exploration. This process contributes to constructing an optimistic vision and increasing readiness and formative and professional career exploration.

## 4. Career adaptability, time perspective, resilience and courage to deal with uncertainty and complexity

As previously stated, one of Life Design interventions’ primary aims is to develop people’s skills to resist, persevere and adapt to a complex and uncertain world (Guichard, 2018). These skills, namely career adaptability, future orientation, resilience, and courage, were proven to play a fundamental role in helping young people face a complex and uncertain present and get back on track towards a positive future. A growing body of literature, as more extensively depicted in this paragraph, also shows these competencies’ crucial role in an unprecedented situation like the post-COVID-19 world.

### 4.1. Career adaptability

Career adaptability is the ability to cope with multiple tasks and changes encountered during formative and professional development (Savickas et al., 2009). Career adaptability comprises four self-regulated internal resources: Concern, Control, Curiosity, and Confidence. The concern is a positive preoccupation with the future and related career envisioning. Control is the ability to be responsible for personal career decisions and build a sense of accountability. Curiosity is the tendency to explore and manage information, selecting and adapting the data about one’s interests. Confidence reflects self-reliance and trust in personal abilities to build efficient plans for the future and cope with career-related obstacles. These attributes are essential for today’s university students facing increasing complexity in life trajectories and challenges in their formative and professional paths. Various research pointed out the crescent need for career counseling to develop career adaptability as a strategy to counteract the COVID-19 pandemic effects on youth’s career construction (Šapale et al., 2021). Moreover, recent research has highlighted the link between career adaptability and other psychosocial constructs such as resilience (Santilli et al., 2020) and courage (Ginevra et al., 2018). All these dimensions contribute to the construction of subjective wellbeing and life satisfaction (Rossier et al., 2012).

### 4.2. Time perspective

Time perspective or future orientation is the human ability to think about the future, anticipating multiple possible scenarios (Nota et al., 2015). According to Lewin (1948), it concerns “plans, aspirations, expectations, and fears concerning events and actions realistically possible in the near or distant future,” comprehending psychological present and past (Trommsdorff et al., 1982). A solid future orientation has been especially highlighted for students and young adults. Future-oriented students had better performance and outcomes at university, higher self-efficacy (e.g., Arian Far et al., 2019), self-esteem, and career success (Praskova and Johnston, 2021). Moreover, studies carried out since the outbreak of COVID-19 have shown how future orientation is positively associated with the use of precautions against the virus and less restrictive rule-breaking behavior (Newson et al., 2022). To conclude, university students with more confidence in their future perspectives are significantly more satisfied with their lives (e.g., Cabras et al., 2017). Higher life satisfaction positively correlates with students’ general wellbeing and plays a significant role in influencing and triggering positive reactions against the obscure context brought out by COVID-19 (Trzebiński et al., 2020).

### 4.3. Resilience

Resilience is the ability to manage life’s negative and challenging experiences, obtaining a positive adaptation (e.g., Masten and Motti-Stefanidi, 2020). Generally, a higher level of resilience is positively related to more positive outcomes, especially in population life satisfaction and wellbeing. The advent of COVID-19 put a strain on population resilience, drastically decreasing its mean level compared to pre-pandemic, regardless of the economic resources possessed (Von Soest et al., 2020). Given its crucial role in times of crisis (e.g., Masten, 2015; Langford et al., 2020; Brivio et al., 2021), resilience’s importance was even recognized at an Institutional level (e.g., DeWit et al., 2020). For example, the Italian National plan for future investments has been named the “National Recovery and Resilience Plan.” As regards students and young people, Folke et al. (2016) have pointed out resilience as the pivotal resource for them to cope and adapt to the constant changes and difficulties that the postmodern world brings. In fact, regarding youth, resilience has been positively correlated with higher school/academic achievement (Rodríguez-Fernández et al., 2018), individual wellbeing, and overall skill level (Gillham et al., 2013). On the other hand, higher resilience means lower stress and anxiety levels, as long as lesser avoidance and other career decision-making difficulties (Shin and Kelly, 2015). A growing body of research highlights the role of resilience as a competence and a protective factor for students during COVID-19. A study by Labrague and De Los Santos (2021) showed that nursing students with higher levels of resilience were less susceptible to the negative psychological effects of the pandemic, such as higher levels of stress and decreased life satisfaction. Many other studies highlighted the importance of promoting resilience in undergraduate and graduate students as a protective factor against stress during the COVID-19 emergency (e.g., Ye et al., 2020). However, according to Der Feltz-Cornelis et al. (2020), interventions to increase resilience should focus on other factors besides reducing psychological stress, such as promoting healthy lifestyles and skills to manage uncertainties. Chen et al. (2021) have shown that resilience plays a central role in reducing levels of anxiety and uncertainty in students in synergy with and through higher levels of future orientation. In line with this, other studies also concerning university students have shown the importance of resilience in maintaining an optimistic, positive, hopeful, and goal-oriented future outlook, preserving and enhancing life satisfaction (Yildirim, 2019).

### 4.4. Courage

From a psychosocial perspective, courage is the ability to persevere in reaching one’s aims and goals despite the subjective sensation of fear (Norton and Weiss, 2009). Courage’s role was more recently highlighted in supporting children (Santilli et al., 2020) and young adults’ career development in complex and changing work conditions, mediating the relation between career adaptability and life satisfaction (Ginevra et al., 2018). Courage was found to positively correlate with “several positive adaptive outcomes and career-related variables, including positive affect, openness, extraversion, positive work behavioral outcomes, and prosocial behaviors” (Ginevra et al., 2018). Despite a growing body of research citing courage as a crucial feature for the post-pandemic world, it mainly focuses on healthcare professionals, undergraduate medicine, and nursing students (e.g., Stephens et al., 2020). Nevertheless, recent findings indicate that courage is one of the strongest predictors of wellbeing and protects against negative emotional states amidst the COVID-19 pandemic (Tee et al., 2021).

## 5. The Life Design Psy-Lab

The Life Design Psy-Lab is a career guidance service established in 2001 within the University of Milan-Bicocca. It offers career guidance across the various stages related to students’ career planning (before matriculation, during their studies, and after graduation), following a Life Design approach (Savickas et al., 2009). To reduce students’ dropout rates and personal uncertainty, Life Design Psy-Lab’s career counsellors deal with a wide variety of career concerns (e.g., “Is my choice the right one?”; “What should I do next?”; “Can I do this?”). In order to meet these needs, Life Design Psy-Lab activities are aimed at developing students’ soft skills, such as tolerance of uncertainty, career adaptability, courage, resilience, and time perspective, as long as satisfaction with one’s life. Through students’ skills development, Life Design Psy-Lab activities’ final goal is to support them in improving different sensibilities, resources, abilities, and, ultimately, proficiency in designing one’s personal and professional life (Annovazzi et al., 2019). In more than 20 years of service, it managed to perform over 12,000 psychological counselling and activities in individual and group settings, face-to-face or online. Since 24 February 2020, due to the COVID-19 emergency and the lockdown, the Life Design Psy-Lab suspended all face-to-face interventions, replacing them with online ones. In particular, a series of online thematic group interventions was organized. Based on the different topics addressed, each thematic group intervention could be composed either of single or multiple meetings, one meeting per week, each meeting lasting from 1 to 2 h. In particular, to better understand the topics addressed and the focuses of this career guidance service, a list of the thematic group interventions organized is herein provided:

- “Why enroll at university in 2020/2021: a plan for the future” dedicated to future enrollees’ formative and life choices through a perspective enhancing and broadening the various planning possibilities in a context of future uncertainty.
- “University at the time of COVID-19: how to plan a choice between constraints and opportunities” to help students in the prefiguration of future educational and professional scenarios in the age of COVID-19.
- “Present for the future, creating new professions to meet new needs” to understand the new professional challenges and encouraging students to open up to innovative and multidisciplinary possibilities/work opportunities.
- “The courage to be yourself: a Life Design training to develop courage within the academic and professional context” aimed at improving the value of courage to face insecurities.
- “Stay or change… this is the question: think and rethink about university choice “to stimulate a reflection about the university choice to make students aware of their expectations, interests, passions, and motivations.
- “Working for others, achieving oneself: professions of high social and environmental importance” focused on the themes of innovation and inclusion to illustrate how social needs can be professionally declined in concrete and contextualized projects.
- “Shaping yourself: a Life Design training on career adaptability” to understand and develop career adaptability.

With special regards to the need of future orientation expressed by students, data collected and the results presented in this paper, the following 4 h intervention will be taken in exam:

- “Future for me, future Why? Who I am and who I want to become” aimed at increasing students’ adaptability and perseverance in complex contexts, restoring a sense of purpose and of trust in their future. This thematic group intervention is the one on which this study’s data are based.

A more detailed description of this intervention, the related procedure and conduction, and its participants, will be provided in the next paragraph.

## 6. Materials and methods

### 6.1. Participants

The sample consisted of 103 students enrolled at the University of Milan-Bicocca who enrolled in a thematic group intervention composed by two meetings. 57.1% of participants are enrolled in Psychology Department, 7.1% in Education Department, 10.7% in Law Department, 17.9% in Economics Department, 3.6% in Sociology Department, and 3.6% in Mathematics Department. Most of the students were female (12 males and 91 females), in line with the national distribution of students’ gender in some departments. In the psychological one, it is equal to 79.9% and in the educational field is 93,8% (AlmaLaurea, 2020). The sample is spread across an age range of 18 to 39 (M = 20.06, SD = 3.37). Finally, the 23.3% of the students also had some work activity. Given the crisis period and following Bicocca’s career guidance services procedures, the Life Design Psy-Lab accepted each student requesting to be enrolled in the intervention.

### 6.2. Procedure

During the COVID-19 pandemic, in November 2021 with regards to this event, the career guidance services of the University of Milano-Bicocca publicized the Life Design Psy-Lab intervention through the website and its social channels. All the students that asked for it were involved in the intervention. Its purpose was explained to the students, and consent and privacy policy forms were obtained. This study followed the ethical rules of the Order of Psychologists of Lombardy and the Italian Society for Vocational Guidance (SIO). The students were free to decline participation in every moment of the career intervention. Students had to fill in the T1 questionnaire with few registry data (e.g., gender, age, etc.) and psychosocial scientific scales assessing skills during the first intervention session (T1). A second questionnaire comprising the same scales and questions about satisfaction with the intervention was filled in after the online intervention (T2). Between T1 and 2 was approximately 1 month.

#### 6.2.1. The intervention

The intervention analyzed in this paper was “Future for me, future Why? Who I am and whom I want to become.” It consisted of two online meetings of approximately 2 h each, performed in December 2021, while Italy was still in state of emergency and the numbers of contagions were rising in the whole nation. The central theme assessed was about proactively opening oneself toward the future, both from a personal and contextual perspective. Four career counselling psychologists performed the intervention from the Life Design Psy-Lab service. During the intervention, students were free to express their questions and reflections, with the career counselling psychologists directly commenting, answering, or, if useful, inviting other participants to join the discussion. They were engaged in practical activities, exercises, and reflections to develop awareness of their career path, exploring new possibilities and opportunities. Activities and exercises performed individually by each student resulted in the construction of a personal portfolio, considered as a final practical result to be kept, consulted and updated at will. Each meeting started with the presentation of the Life Design Psy-Lab career guidance service, the introduction of the career counsellors involved, and the intervention aims. Before the beginning of the first meeting, the T1 questionnaire was administered to each student who gave informed consent to data acquisition and analysis. The first meeting was more extensively focused on personal training and reflection about an aware personal life design. Students were encouraged to imagine and accept the future as a part of everyone’s present life, considering it as something to cherish and not to be feared. In particular, in the first meeting there were three activities, each lasting approximately 15 min. The first activity, named “I am…my starting line!” asked each student to complete the statement “I am…” in every way they could think of, freely using words and images. This activity aimed to gain awareness of the complexity and richness of one’s life and the tendency to over-simplify it. The second activity was called “My skills to face difficulties” and asked each student to express each competence, resource, skill and ability they possessed. They could also link these skills to the difficulties they eventually faced. Before performing this activity, students were stimulated to reflect on the difference between soft and hard skills and provided with some examples. The aim was to develop strategies to overcome difficulties and deal with complexity based on everyone’s experience. The third activity, called “My goals, my future,” invited students to focalize their shortest, short, medium and long-term goals, taking inspiration from what they did in the previous activities.

In the second meeting, the focus shifted from an individual to a more contextual one. In this meeting, students reflected on the challenges posed by the present global context and the opportunity to build a preventive approach to face problems and doubts. The first activity was called “The context we live in” and asked students to build a representation of their life context using words, images, and photos at will. The second activity was called “The word as we know it.” Students answered a series of questions about the world’s context (e.g., about world poverty rates, instruction level, etc.) in order to become aware of their more or less optimistic or pessimistic representation of the world. The last activity was called “Goals and future scenarios.” It challenged students to identify their favorite Sustainable Development Goal (United Nations, 2015) and to think about how to make it real, linking it to one of the goals they chose for themselves at the end of the first meeting. At the end of the meeting, the questionnaire (T2) was re-administered.

### 6.3. Measures

#### 6.3.1. Career adapt-abilities inventory

Career Adapt-abilities Inventory (CAAI; Soresi et al., 2012) was used to measure career adaptability. It consists of 24 items on a 5-point Likert-type scale from 1 (not strong) to 5 (strongest). The questionnaire is divided into 4 dimensions: Concern, Control, Curiosity, and Confidence. The Concern dimension investigates the propensity to positively care about one’s future (e.g., “Becoming aware of the educational and vocational choices that I must make”). The second factor, Control, explores the propensity to take responsibility for the choices one makes (e.g., “Taking responsibility for my actions”). The Curiosity factor considers the propensity to be curious and explore new opportunities and possibilities; for example, one item is “Investigating options before making a choice.” Finally, the last dimension regards the propensity to have self-confidence, for instance, “Learning new skills.” These dimensions, when combined, form a total score that shows ad individual’s level of career adaptability. For the present study, the Cronbach’s alpha corresponds to 0.79 for the Concern, 0.78 for Control, 0.81 for Curiosity, and 0.84 for Confidence.

#### 6.3.2. Design my future

Design My Future (Santilli et al., 2017) is a 19-item scale with a 5-point Likert scale response (1 = It does not describe me at all; 5 = It describes me very well). This scale is composed of two subscales: Future Orientation and Resilience. In particular, the Future Orientation is composed of 11 items, such as “I often think about building a positive future for myself”; while Resilience consists of 8 items, for example, “I do not get discouraged very easily after a failure.” In the present study, Cronbach’s alpha, respectively, corresponds to 0.89 and 0.82.

#### 6.3.3. Courage

The scale used in this study refers to the mono-factorial version suggested by the studies of Howard and Alipour (2014) and is based on the reduced version proposed by Norton and Weiss (2009). Specifically, we used the Italian version of the scale, which was validated in a study conducted by Ginevra and colleagues in 2020. The scale consists of 6 items on a 7-point Likert scale from 1 (never) to 7 (always); an example of an item is: “I tend to face my fears” or “If I am worried or anxious about something, I will do or face it anyway.” For this sample, Cronbach’s alpha corresponds to 0.74.

#### 6.3.4. Satisfaction with life scale

This scale (Diener et al., 1985) assesses satisfaction and overall quality of one’s life using 5 items on a 7-point Likert scale from 1 (Strongly disagree) to 7 (Strongly agree); an example of an item is “I am satisfied with my life.” In the present study, we use the Italian version of this scale: for this sample, Cronbach’s alpha corresponds to 0.84.

## 7. Data analysis

Prior to testing the effect of the intervention, several preliminary analyses were conducted. First, missing responses and skewness and kurtosis were assessed. Second, the normality of distributions of career adaptability, courage, resilience, time perspective, and life satisfaction were tested using Kolmogorov–Smirnov statistic. Third, correlations were run to examine the relationship between career adaptability, courage, resilience, time perspective, and life satisfaction. Repeated measures analyses of variance (ANOVAs) were performed to compare the mean pre-and post-test scores of the four CAAI constructs, resilience, time perspective, courage, and satisfaction in life. Regarding the effects of participation in the formative training presented in this study, the levels of career adaptability, its components (Concern, Control, and Curiosity), satisfaction in life, time perspective, resilience, and courage were analyzed with a one-way repeated measures ANOVA. Given the presence of only two levels (T1 and T2), Greenhouse–Geisser, Huynh-Feldt, and assumptions of Mauchly’s test of Sphericity are assumed.

## 8. Results

In this paragraph, results from the intervention performed will be showed. These results stem from the T1 and T2 questionnaires filled in, respectively, prior to the intervention and at its end.

Table 1 shows the correlations among the study variables in the pre- and post-test.

**Table 1:** Correlations among the study variables at pre- and post-test.

Measure	1	2	3	4	5	6	7	8	9
1. Concern	–	0.648^**^	0.736^**^	0.711^**^	0.878^**^	0.428^**^	0.683^**^	0.573^**^	0.432^**^
2. Control	0.521^**^	–	0.670^**^	0.758^**^	0.870^**^	0.569^**^	0.621^**^	0.721^**^	0.567^**^
3. Curiosity	0.646^**^	0.536^**^	–	0.755^**^	0.888^**^	0.500^**^	0.684^**^	0.629^**^	0.503^**^
4. Confidence	0.675^**^	0.636^**^	0.625^**^	–	0.907^**^	0.563^**^	0.595^**^	0.726^**^	0.603^**^
5. Career Adaptability	0.849^**^	0.804^**^	0.834^**^	0.871^**^	–	0.581^**^	0.730^**^	0.747^**^	0.593^**^
6. Satisfaction in Life	0.272^**^	0.427^**^	0.387^**^	0.444^**^	0.454^**^	–	0.517^**^	0.516^**^	0.392^**^
7. Time Perspective	0.464 ^**^	0.440^**^	0.544^**^	0.416^**^	0.556^**^	0.292^**^	–	0.601^**^	0.460^**^
8. Resilience	0.455^**^	0.681^**^	0.524^**^	0.609^**^	0.675^**^	0.454^**^	0.502^**^	–	0.679^**^
9. Courage	0.355^**^	0.550^**^	0.538^**^	0.525^**^	0.585^**^	0.364^**^	0.418^**^	0.639^**^	–

Note: the intercorrelations for the pre-test (*n* = 108) are presented below the diagonal, and the intercorrelations for post-test (*n* = 108) are presented above the diagonal.

* *p* < 0.05.

** *p* < 0.01.

The correlation matrix showed that a higher level of Career Adaptability is related to a higher level of resilience, time perspective, and courage. These results mean that improving career adaptability in students means having an impact on the capacity to face difficulties and complexity, with perseverance despite fear, actively constructing future career plans. Moreover, the level of all personal resources is related to satisfaction with personal life, which means that working on improving skills means working for life satisfaction. The analysis revealed that the students that attended the career intervention showed a significant increase of Control, Concern, and Curiosity, but not for Confidence, and in general, a higher level at T2 of the total score of Career Adaptability. Indeed, there was a significant effect of participation in the training on Career Adaptability *F* (1, 102) = 12,1, *p* < 0.001 and its components: Control *F* (1, 102) = 7,54, *p* < 0.05, Concern *F* (1, 102) = 10,43, *p* < 0.05, Curiosity *F* (1, 102) = 6,92, *p* < 0.05. A comparison between main scores at different time points shows a significant difference at T1 and T2, *F* (1, 102) = 21,57, *p* < 0.01 on time perspective. Specifically, the average levels of future orientation were lower in the pre-test than in the T2 questionnaire. Moreover, there was a significant difference *F* (1, 102) = 15,53, *p* < 0.001 between T1 and T2 in resilience. Specifically, the average levels of the capacity to face life’s negative and challenging experiences, obtaining a positive adaptation, were higher in the post-test.

There was a significant main effect of participation in the training on courage *F* (1, 102) = 11,57, *p* < 0.001. Mainly, the post-test showed a higher level of ability to persevere in reaching one’s aims and goals despite the subjective sensation of fear. Finally, there was a significant difference *F* (1, 102) = 14,70, *p* = 0.001 between T1 and T2 in satisfaction in life. Table 2 shows the means and standard deviations for the participants of the Life Design Psy-Lab intervention for the pre-test and post-test.

**Table 2:** Means and standard deviations at pre- and post-test.

Measure	*M Pre-test*	*SD Pre-test*	*M Post-test*	*SD Post-test*
1. Concern	3.53	0.65	3.69	0.69
2. Control	3.70	0.64	3.82	0.68
3. Curiosity	3.53	0.62	3.66	0.61
4. Confidence	3.66	0.60	3.73	0.62
5. Career adaptability	3.61	0.53	3.73	0.57
6. Satisfaction in life	4.30	1.10	4.55	1.08
7. Time perspective	3.41	0.70	3.62	0.70
8. Resilience	3.22	0.65	3.40	0.67
9.Courage	4.24	0.88	4.46	1.01

Overall, participants evaluated the Life Design Psy-Lab intervention fundamental to reflect about themselves and the world: “It was helpful to reflect about our opportunities and our future” (Participant #13). A total of 33.3% of participants indicated that they were totally satisfied with the intervention, and 50.4% were very satisfied. 9.4% were medium satisfied, 6.9% a little satisfied, and none was not satisfied at all.

## 9. Discussion

This study aimed to assess the effectiveness of an online group career counselling intervention in university students during the COVID-19 emergency, carried out by the Life Design Psy-Lab academic career guidance service. The intervention’s goal was to increase students’ abilities to face the challenges and difficulties of the postmodern era, corroborated by the COVID-19 emergency. During the pandemic, the service became a reference point for students, even when every other situation for ‘physical’ presence and traditional interpersonal relationships was temporarily missing. The Life Design Psy-Lab intervention, starting from a realistic reading of the current and future social and professional reality, was able to foster in students the construction of conscious decision-making processes in line with the national emergency and current difficulties. It has encouraged reflection on strategies for managing the mounting fear of the future precepted by its users. Comparing the participants’ results in the self-report questionnaires administered before and after the intervention shows a statistically significant increase in their career adaptability, resilience, future orientation, and courage, as well as their overall satisfaction with life. These results should be contextualized and read in terms of the two main problematic processes faced by students and, consequently, by career counsellors. On the one hand, the complex labor market and frequent job transitions characterize contemporaneity. They call for flexible young adults who can express themselves and their precious ideas and abilities in multiple complex contexts. On the other hand, this pre-existing scenario met the health, economic and social crisis catalyzed by the COVID-19 pandemics. This combination hindered people’s aspirations and ability to get engaged to achieve their goals, overcome difficulties, and proceed to their desired future. In this context of uncertainty, in line with Maree (2021) and Šapale et al. (2021), increasing our participants’ adaptability helped them start or re-start walking their present and future formative and professional paths. Career adaptability also fosters an active choice aptitude and an openness to new opportunities against a feeling of heaviness and powerlessness against an unstable present and an uncertain future. It contributes to a shift from a sense of purposeless and exhausting “worry” to a more purposeful sense of concern, leading to preventive actions to safeguard one’s interests, dreams, and desires. Nevertheless, as stated by Mahmud et al. (2021), one of the heaviest consequences suffered by young people linked to the present pandemic context is a reduction of one’s time perception to a never-ending present, in which future dreams, desires, and aspiration are numbed by social isolation, unpredictability, or find no place at all. For this reason, the intervention led the participants into a guided exploration of themselves, their aspirations, desires, and precepted difficulties. Also, in line with the challenges to Life Design Counseling depicted by Wen et al. (2020), this study contributed to answer the ask for career guidance practices and interventions performed by specialized career counsellors and capable of fostering career adaptability using online training. Great importance was given to stimulating ideas about their future and short-, medium-and long-term desired goals. In this same direction, reactivating the ability to think positively about one’s future is also an important achievement, concretely seen in our participants’ increase in their time perspective. In line with Trzebiński et al. (2020), for a university student to consider their future as a resource and a motivational drive, rather than a fearful problem to be solved, is essential to proceed optimistically and hopefully with their life, especially in this COVID-19 period, obtaining academic and professional success. Also, the students who enrolled in the Life Design Psy-Lab’s intervention were encouraged to reflect on and face the difficulties they were experimenting with. They were guided into analyzing and decomposing their struggles into more comprehensible ones. This way, they could get back the power and energy to get into action and walk towards their goals one step after another. This was testified by an increase in their courage skill, which is promptly aimed at achieving one’s desired achievements, despite the difficulties one has to face and will encounter. It is an aptitude of not giving up, maintaining the due importance to one’s desires, which is linked to Hope for one’s future and stronger self-esteem (Ginevra et al., 2020). Regarding another skill strongly linked with managing life’s negative and difficult experiences, the Life Design Psy-Lab intervention participants saw their precepted level of resilience significantly increase after participating in the proposed activities. As mentioned before, we suggested and guided the participants in exploring their difficulties, as long as their precepted skills not only to overcome them. Psychological resilience is linked to a more positive adaptation to a hard situation, like the COVID-19 crisis. It is not just about resisting the difficulties but about finding a way to exploit them to obtain personal growth, leading towards more optimistic and hopeful visions and pathways for the future and increasing one’s satisfaction with their life (Yildirim, 2019). An increase in students’ general satisfaction with life is the final topic we want to highlight in this paper. The COVID-19 period hindered young adults’ lives from several points of view, leading to a general diminishing in life satisfaction. Each aspect increased by the presented intervention, namely adaptability, resilience, courage, and future orientation, is positively linked with this central aspect of human life. Therefore, we can conclude that the online Life Design Psy-Lab intervention contributed to construct a response to students’ emerging needs in a complex world with an uncertain future. This may be considered a step forward in career guidance’s ultimate challenge in the COVID-19 era, which is asking this field’s professionals “how innovative and contextualized career counselling based on counselling for self-and career construction and Life Design principles can meet young people’s need for e-career counselling during and probably after the COVID-19 pandemic” (Maree, 2021).

## 10. Limitations and future research perspective

Although some interesting results emerged, this study presents some limitations. The first one is the absence of a control group. It is theoretically possible that the changes in students are due to the mere passage of time or autonomous development. Following Maree (2019), future studies should use a quasi-experimental design with alternative treatment and no-treatment control groups. However, we believe these explanations for student changes are highly unlikely as T1 and T2 occurred during the pandemic COVID-19. In such a difficult period, it is unlike that students autonomously started a deep self-reflection about their future, modifying the levels of personal resources. Indeed, in line with the study of Hatzichristou et al. (2017), changes in psychological skills to face a complex crisis may be more expectable by those who carry out psychological sessions. The second limit concerns that the findings and reflections only refer to the north Italian university context. The following research should involve participants from other contexts to verify the possibility of generalizing the results. Also, related to this, our sample presents a gender distribution imbalance. As more extensively depicted in the sample paragraph, the majority of the students that participated to the interventions were from the psychology department, which itself has an unbalanced gender distribution. The third limit pertains that the effectiveness of the activities was only measured once after the program’s conclusion. Future research could consider implementing a 6- and 12-month follow-up intervention for qualitative and quantitative measures. Despite the difficulties, these limits have undoubtedly to be overcome in the future.

## Data availability statement

The raw data supporting the conclusions of this article will be made available by the authors, without undue reservation.

## Ethics statement

The studies involving human participants were reviewed and approved by Commissione per la Valutazione della Ricerca (CRIP; Commission for the Research Evaluation) - University of Milano Bicocca. The patients/participants provided their written informed consent to participate in this study.

## Author contributions

CA: conceptualization (lead), writing – original draft (lead), methodology and formal analysis (lead), and writing – review and editing (equal). EC: coordination (lead), conceptualization (equal), and review and editing (supporting). MS: methodology and formal analysis (supporting), writing – original draft (equal), and writing – review and editing (equal). RR: writing – original draft (equal) and writing – review and editing (equal). PG: writing – original draft (supporting) and writing – review and editing (supporting). DM: conceptualization (equal) and writing – original draft (supporting). All authors contributed to the article and approved the submitted version.

## Conflict of interest

The authors declare that the research was conducted in the absence of any commercial or financial relationships that could be construed as a potential conflict of interest.

## Publisher’s note

All claims expressed in this article are solely those of the authors and do not necessarily represent those of their affiliated organizations, or those of the publisher, the editors and the reviewers. Any product that may be evaluated in this article, or claim that may be made by its manufacturer, is not guaranteed or endorsed by the publisher.
